# Integrating Findings Into Practice: Assessing External Validity of Congestive Heart Failure Trials

**DOI:** 10.14740/cr2191

**Published:** 2026-04-15

**Authors:** Lydia Hashemi, Ryan Langerman, Chance Bratten, Adam Khan, Alec Young, Taylor Gardner, Eli Paul, Gershon Koshy, Alicia Ito Ford, Matt Vassar

**Affiliations:** a Office of Medical Student Research, Oklahoma State University Center for Health Sciences, Tulsa, OK, USA; b Department of Medicine - Cardiology, Oklahoma State University Medical Center, Tulsa, OK, USA; c Department of Psychiatry and Behavioral Sciences, Oklahoma State University Center for Health Sciences, Tulsa, OK, USA

**Keywords:** Congestive heart failure, Randomized controlled trial, Patient-centered outcome, Pragmatic clinical trials, Research transparency, External validity

## Abstract

**Background:**

Congestive heart failure (CHF) remains a major global health issue, affecting millions of adults worldwide, contributing to significant hospitalizations and mortality. While randomized controlled trials (RCTs) are essential for improving CHF care, their external validity remains uncertain. This study evaluates the external validity of CHF RCTs published between 2014 and 2024 using the criteria developed by van ’t Hooft et al.

**Methods:**

A systematic appraisal of CHF RCTs was performed using MEDLINE and Embase on April 1, 2025. Included studies were full-text, English-language, human trials focused on CHF interventions. Trials were assessed with a 13-criterion guideline covering pragmatic principles, context, information gain, feasibility, transparency, value, and patient-centeredness. Two reviewers independently screened and extracted data, resolving discrepancies by consensus. Trial characteristics and predictors of usefulness were analyzed via linear regression and descriptive statistics.

**Results:**

Among 659 records screened, 44 met inclusion criteria. Of these, 15.9% demonstrated information gain, 36.4% provided context placement, and 4.5% avoided violations of pragmatic principles. Patient-centeredness was fully addressed in 54.5% of trials and 38.6% demonstrated feasibility. A majority of studies disclosed funding at 77.3% and conflict of interest statements at 68.2%. Transparency and usefulness values showed a modest upward trend over time (r = 0.42, P < 0.05).

**Conclusions:**

Although CHF RCTs are the gold standard for evaluating new interventions, many fall short in pragmatism, information value, power analysis, and data transparency. Future trials may benefit from prioritizing pragmatic principles, adequate power calculations, cost analysis, and data sharing.

## Introduction

Congestive heart failure (CHF) affects an estimated 64.3 million adults worldwide, contributing to about a million hospitalizations annually [1, 2]. Many effective interventions exist such as pharmacologic therapies (β-blockers and angiotensin-converting enzyme inhibitors) and medical devices (implantable cardioverter-defibrillators, cardiac resynchronization therapy) [3]. Randomized controlled trials (RCTs) are key to demonstrating clinical effectiveness, yet their primary endpoints do not always match clinicians’ needs. Historically, some CHF RCTs investigating interventions have used surrogate endpoints—biomarkers, increased left ventricular ejection fraction, improved 6-min walk distance—as their primary outcomes [4–7]. Surrogate outcomes can be useful in comparing interventions, but are not considered to be patient-centered [7]. Patient-centered outcomes such as symptom burden, hospitalizations, and quality of life can be of greater benefit when evaluating therapeutic efficacy. Additionally, RCTs apply exclusion criteria that further restrict the applicability of trial findings to routine practice.

In order for RCTs to be relevant to clinicians, participants in trials should be representative of the general population of people with CHF. Yet many trial protocols exclude patients with common comorbidities: older adults; those with hypertension; individuals with chronic obstructive pulmonary disease, chronic kidney disease, or diabetes; and those classified as New York Heart Association class III-IV [2, 8–12]. Excluding or not reporting baseline comorbidities raises the concern of trial generalizability [11]. The use of ideal patients rather than real-world patients limits the ability of patient outcomes from such trials to be applied clinically.

These exclusions can diminish RCT applicability, leaving clinicians to rely on trials that do not reflect their patients. To assess whether RCTs are clinically useful, van ’t Hooft et al proposed a usefulness criterion in 2023 [13]. This criterion comprises eight domains encompassing 13 items that index methodological rigor, reporting transparency, and pragmatic relevance. Applying this tool can help identify gaps in study design, trial limitations, and lack of generalizability. This criterion has been applied to preterm birth and pediatrics, but has not yet been applied to CHF [13, 14]. To assess whether RCTs are clinically informative, van ’t Hooft et al proposed a usefulness framework in 2023 designed to evaluate whether trials generate evidence that can meaningfully inform clinical decision-making. This framework incorporates pragmatic design, contextualization within existing evidence, feasibility, patient-centered outcomes, and research transparency to evaluate the broader clinical relevance of trial findings beyond traditional measures of internal validity. Although some elements of the criteria require interpretive judgment, the framework was developed based on established methodological principles and has been applied in prior meta-research studies evaluating randomized trials in other clinical fields. Applying this structured framework allows investigators to systematically identify aspects of trial design and reporting that may influence the translation of research findings into routine clinical practice. To our knowledge, this framework has not yet been applied to RCTs evaluating interventions for CHF.

## Materials and Methods

### Reproducibility and study design

This study evaluated the usefulness of RCTs investigating interventions for CHF. Eligible studies were those published (electronically or in print) in full-text from January 1, 2014, to December 31, 2024. Before formal data collection began, the search strategy, eligibility criteria, and data extraction form were tested on a sample of five articles. The study conformed with the Preferred Reporting Items for Systematic Reviews and Meta-Analysis (PRISMA) 2020 guidelines and was publicly preregistered on Open Science Framework (OSF) to ensure both transparency and reproducibility [15]. All items, including data collection forms, data dictionary, study protocol, raw data, and analysis scripts are publicly accessible in our OSF repository [16].

### Search strategy

**Figure 1:**
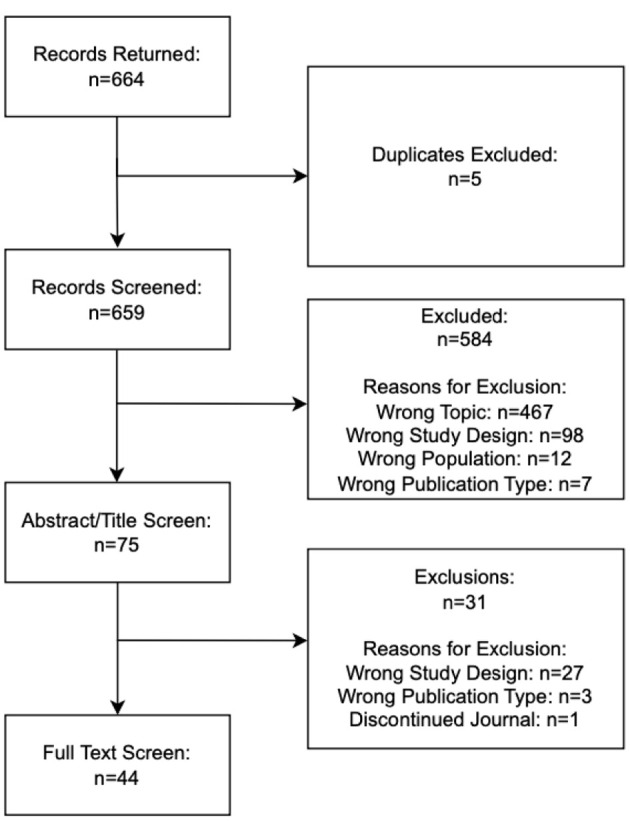
PRISMA flow diagram.

A systematic literature search was conducted on May 13, 2025, using Embase via Elsevier, which includes simultaneous access to MEDLINE. The strategy combined controlled vocabulary and keyword terms relevant to CHF, RCTs, and interventions. Limits were applied to include only studies involving human subjects, published in English, available in full-text, and published from January 1, 2014, to December 31, 2024. The complete search strategy is available on OSF. RCT search filters were adopted from Cochrane strategies [17]. No additional sources such as trial registries, citation tracking, gray literature, or author contacts were used. Relevant search results were imported into the screening tool Rayyan [18]. Duplicates were removed using the platform’s deduplication tool, and two reviewers (LH, RL) individually assessed titles and abstracts for eligibility. Disagreements were settled through discussion or resolved by a third reviewer (CB). The final number of articles retrieved from each database is included in Figure 1.

### Training

All team members received standardized training covering the study’s goals, inclusion and exclusion screening criteria, and the data extraction procedure, with particular attention to the van ’t Hooft usefulness criteria. Live instruction was led by faculty researchers (MV, AF). Calibration sessions using five CHF RCTs were conducted to ensure consistency. Reviewers (LH, RL) were also trained in the use of Rayyan and a customized, pilot-tested Google Form used for data extraction. Disparities during the pilot phase were resolved by group collaboration and discussion. Written guidelines were used to aid uniform decision-making during data extraction.

### Eligibility criteria/screening

Articles included in the study were RCTs assessing interventions for CHF with a primary focus on clinical outcomes. The inclusion criteria required full-text RCTs, published electronically or in print, from January 1, 2014, to December 31, 2024, in English, involving human subjects aged 18 or older with CHF who received an intervention (e.g., pharmacological, procedural, or behavioral). The first full report of the trials’ primary outcomes was included. Secondary analyses (e.g., subgroup, exploratory, or mediation) published separately were excluded. Trials were excluded if their primary focus was not CHF in adults, or if they were non-randomized studies, pharmacokinetic/pharmacodynamic-only trials, single-group studies, studies reporting only safety outcomes, or non-primary research such as reviews, meta-analyses, case series, and editorials. Two reviewers (LH, RL) independently screened all abstracts and titles based on these criteria, followed by a full-text screening of the rest of the articles. Any disagreements were settled through consensus or consultation with a third reviewer (CB).

### Data extraction

Data from eligible trials were independently extracted in duplicate by two reviewers (LH, RL) using a standardized, pilot-tested Google Form. Extracted variables included study title, PubMed ID, journal name and specialty, impact factor, publication year, trial registry number, continent(s), intervention type, sample size, funding source, and trial phase. Each RCT was assessed for usefulness using the 13-item criteria developed by van ’t Hooft et al, with each criterion valued as 0 (not met), 1 (partially met), or 2 (fully met), yielding a total value between 0 and 26. The criteria are defined in Table 1. These guidelines were adapted from previous studies to ensure consistency and structure in our assessment [19, 20]. To enhance consistency and resolve discrepancies, two data extractors (LH, RL) individually piloted the extraction on five sample RCTs. During the full-text data extraction process, all criteria were evaluated independently and in duplicate, with one deviation: the “problem base” criterion. This criterion was evaluated by a clinical investigator (GK), due to the need for clinical and contextual understanding in order to decide which trials addressed topics with wide patient relevance and significant health impact. Disagreements were settled by consensus or with input from a third reviewer (CB). This approach is consistent with other meta-research studies applying structured evaluation frameworks to assess methodological and reporting practices in clinical trials [21].

**Table 1:** Usefulness Criteria Item Descriptions

Clinical utility item	Description
1. Problem base	Health impact depends on prevalence, individual burden, and cost, including common and rare diseases.
2. Context placement	Clinical research is most useful when informed by systematic reviews that integrate new findings with existing evidence.
3. Information gain	Informative clinical research requires adequately powered studies focused on critical outcomes, avoiding surrogate measures, and integrating results with prior evidence for meaningful practice changes.
4. Pragmatism	Pragmatic research evaluates the real-world effectiveness of interventions, emphasizing generalizability and practicality over controlled, ideal conditions.
5. Patient-centeredness	Patient-centered research prioritizes questions and outcomes that matter most to patients, involving them in setting priorities and developing core outcomes.
6. Value for money	Value-for-money research evaluates the expected benefits of a study against its costs, using analyses like value-of-information and budget impact to guide design and funding decisions.
7. Feasibility	Feasibility in research depends on realistic recruitment goals, adequate power, and collaborative networks to overcome challenges like patient availability and overestimated sample sizes.
Transparency item	Description
8A. Preregistration	Preregistration is the process of registering a study in an official registry database before recruiting the first patient.
8B. Public protocol	The protocol and data analysis plan should be publicly available before the trial starts and before data analysis.
8C. Protocol adherence	Any deviations from the original protocol should be justified, updated in the protocol, and reapproved by the ethics committee, with modification statements included in the manuscript.
8D. Funding statement	Funding sources for trial conduction should be clearly stated.
8E. Conflict of interest statement	Conflicts of interest should be clearly and completely disclosed.
8F. Data availability	Freely available raw data, including statistical code and output, is becoming the norm to ensure transparency, facilitate meta-analyses, and address concerns about study trustworthiness.

We defined clinical utility as the extent to which an RCT provides clinically relevant, generalizable, and reproducible information. Trials with high clinical utility produce results that reflect patient priorities, can be applied to diverse patient populations, align with existing evidence, and evaluate outcomes that significantly influence diagnosis, treatment, or patient well-being. Transparency was defined as the openness, completeness, and reproducibility of RCT design, conduct, and reporting. Transparent trials allow independent verification, minimize the risk of selective reporting, and contribute to the broader research ecosystem by enabling replication, meta-analysis, and critical appraisal. Overall usefulness integrates these two concepts.

### Data analysis

All statistical analyses were performed using R (version 4.3.1) in RStudio (version 2023.09.1+494). Descriptive statistics summarized study characteristics and usefulness values, using frequencies, percentages, medians, and interquartile ranges (IQRs) as appropriate. Each trial was assigned a total usefulness value by combining its 13 individual criteria values, which were separated into two categories: transparency and clinical utility. Linear regression models were used to assess the relationship between publication year and usefulness values, as well as between year of publication and clinical utility or transparency values separately. Pearson correlation coefficients (with significance set at P < 0.05) quantified the relation between clinical utility values and transparency. All analyses and code were made publicly available via OSF to ensure transparency of data and reproducibility.

### Ethical oversight

Our research protocol was submitted to the Oklahoma State University Center for Health Sciences Institutional Review Board (IRB) (IRB # 2025062) for review and was determined to not meet the regulatory definition of human subjects research in accordance with the Code of Federal Regulations (45 CFR 46.102(d) and (f)) [22].

### Protocol deviations

Two deviations from the preregistered protocol occurred. First, the planned risk of bias assessment using the Cochrane RoB 2.0 tool with ChatGPT support was removed. This was because ChatGPT was not designed for this task. Our intended alternative, RobotReviewer, was no longer operational. Second, the “problem base” criterion was assessed by a clinical investigator (GK) rather than in duplicate, due to the need for clinical and contextual expertise. All other procedures followed the original protocol and are documented in the OSF registry.

## Results

### Identification and inclusion of eligible studies

Our initial search on Embase yielded 664 records. After identifying and excluding five duplicates, the pool was reduced to 659 unique records. Of these, 584 were excluded based on eligibility criteria. After reviewing the full-text of the remaining 75 RCTs, 31 additional RCTs were excluded, and 44 RCTs were included in our final analysis. Explanations for exclusions at each stage of screening are detailed in Figure 1.

### Trial demographics and publication characteristics

The most prevalent journal categories among the 44 trials were cardiology (50%; 22/44) and general medicine (38.6%; 17/44). The median impact factor of the journals publishing the included trials was 3.0 (IQR, 2–6). The majority of trials were conducted in Asia (47.7%; 21/44), followed by Europe (18.2%; 8/44). The median sample size across trials was 82 participants (IQR, 52.0–191). Regarding funding, 38.6% (17/44) of studies were privately funded, while 27.3% (12/44) did not report any funding source. The publication years of our trials were dispersed somewhat evenly between 2013 and 2024, with the most (18.2%; 8/44) being published in 2014. The general characteristics of our study are summarized in Supplementary Material 1 (cr.elmerpub.com).

### Evaluation of trial clinical utility and transparency

Data showed that the majority of trials performed well in disclosure of funding, while data sharing, pragmatism, and economic evaluations standards were less frequently met. The most commonly met criterion was disclosure of funding, with 77.3% (34/44) of trials providing funding statements. In opposition, most studies were not pragmatic, with only 4.5% (2/44) avoiding the violation of pragmatic principles. A pre-trial cost analysis was only reported in one study at 2.3% (1/44). Further, 77.3% (34/44) of trials failed to make their data publicly accessible. As far as transparency in protocol, 70.5% (31/44) of trials failed to publish their protocol. Table 2 shows the full utility and transparency results.

**Table 2:** Criteria Findings

Clinical utility criteria	N = 44	Transparency criteria	N = 44
Problem base, n (%)		Preregistration, n (%)	
Full	4 (9.1)	Full	18 (40.9)
Partial	25 (56.8)	Partial	9 (20.5)
Absent	15 (34.1)	Absent	17 (38.6)
Context placement, n (%)		Public protocol, n (%)	
Full	16 (36.4)	Full	6 (13.6)
Partial	28 (63.6)	Partial	7 (15.9)
Absent	0 (0.0)	Absent	31 (70.5)
Information gain, n (%)		Adherence to protocol, n (%)	
Full	7 (15.9)	Full	12 (27.3)
Partial	4 (9.1)	Partial	0 (0.0)
Absent	33 (75.0)	Absent	32 (72.7)
Pragmatism, n (%)		Funding stated, n (%)	
Full	2 (4.5)	Full	34 (77.3)
Partial	3 (6.8)	Partial	0 (0.0)
Absent	39 (88.6)	Absent	10 (22.7)
Patient centeredness, n (%)		COIs, n (%)	
Full	24 (54.5)	Full	30 (68.2)
Partial	6 (13.6)	Partial	2 (4.5)
Absent	14 (31.8)	Absent	12 (27.3)
Value for money, n (%)		Raw data, n (%)	
Full	1 (2.3)	Full	6 (13.6)
Partial	1 (2.3)	Partial	4 (9.1)
Absent	42 (95.5)	Absent	34 (77.3)
Feasibility, n (%)			
Full	17 (38.6)		
Partial	3 (6.8)		
Absent	24 (54.5)		

### Evaluation of context placement, feasibility reason, and pragmatism violation

A total of 62 pragmatic violations were seen across 44 RCTs. The most common pragmatic limitations observed were design features that may reduce real-world applicability, including extensive blinding procedures (50%; 22/44) and the use of placebo controls (40.9%; 18/44). Only three (6.8%) RCTs avoided pragmatic violations. At least one systematic review was cited in 40.9% (18/44) of RCTs. Further, 40.9% (18/44) of RCTs were deemed feasible based on their calculation of a sample size and their recruitment planning. Lastly, 45.5% (20/44) of RCTs were deemed non-feasible, mostly due to a lack of power calculation. For more details regarding systematic review inclusion, feasibility, and pragmatism (Table 3).

**Table 3:** Systematic Review Inclusion, Feasibility, and Pragmatism in Clinical Trials

Characteristic	N = 44
Prior systematic review, n (%)	
Not cited or performed	26 (59.1)
Systematic review cited	18 (40.9)
Non-feasibility reason, n (%)	
No reason provided	20 (45.5)
Study was feasible	18 (40.9)
Attrition	3 (6.8)
Low recruitment speed	3 (6.8)
Pragmatism violation^a^, n (%)	
Blinding of assessors	22 (50.0)
Placebo-controlled	18 (40.9)
Employs a new intervention	12 (27.3)
Single-centered trial	9 (20.5)
No pragmatism violation	3 (6.8)
Employs a new indication	1 (2.3)

^a^Multiple studies had more than one pragmatism violation, resulting in a total N = 65 for this section.

### Changes in clinical utility, transparency, and total usefulness across publication years

**Figure 2:**
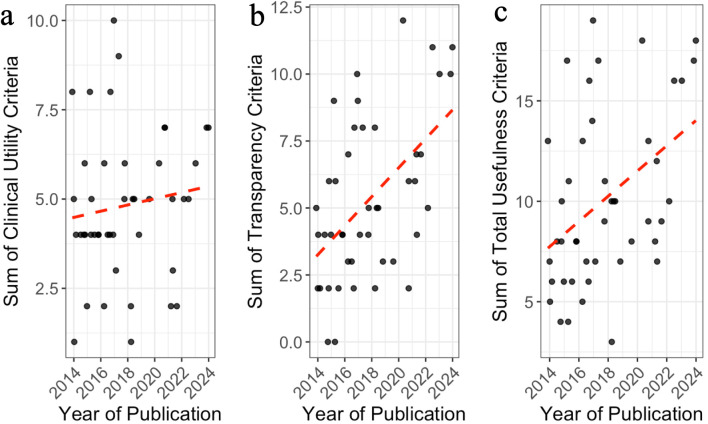
Trends in clinical utility, transparency values, and total usefulness of randomized controlled trials (RCTs) on congestive heart failure (CHF) by year of publication. Panels (a)–(c) display the distribution of summed values for clinical utility criteria (a), transparency criteria (b), and total usefulness (c) across publication years. Each dot represents an individual RCT. Red dashed lines represent fitted linear regression lines.

Figure 2 illustrates yearly trends in summed clinical utility, transparency, and total usefulness for CHF RCTs. Clinical utility climbed modestly, with most values ranging between 3 and 7 (Fig. 2a). Transparency rose more sharply, reflecting better protocol availability, registration, and data sharing (Fig. 2b). Together, these shifts produced a clear rise in total usefulness, driven mainly by transparency gains (Fig. 2c). These trends suggest that while clinical utility remains relatively stagnant, recent CHF RCTs are increasingly adopting more transparent practices, contributing to an overall improvement in trial usefulness over the past decade.

### Correlation between transparency and clinical utility

**Figure 3:**
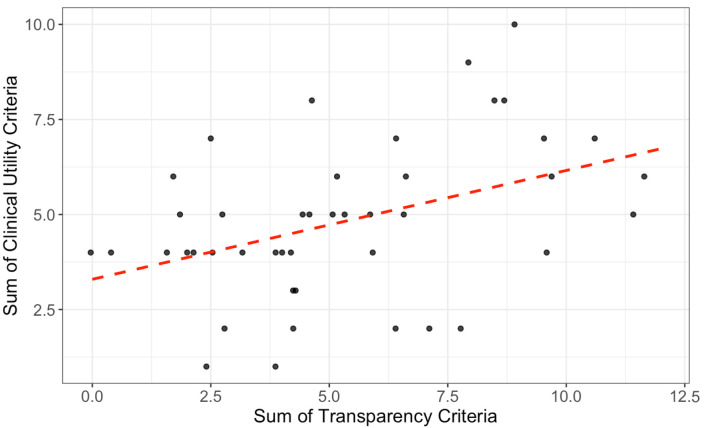
Each point represents a randomized controlled trial (RCT) assessing interventions in congestive heart failure (CHF). The red dashed line indicates a fitted linear regression. A positive, statistically significant correlation was observed between transparency and clinical utility values (Pearson’s r = 0.42, 95% CI: 0.14–0.64, t(42) = 3.03, P = 0.004).

There was a positive correlation between transparency and clinical utility values across all trials. As illustrated in Figure 3, trials demonstrating higher levels of transparency generally exhibited greater clinical utility. This relationship was statistically significant and moderately strong, with a Pearson correlation coefficient of r = 0.42 (95% CI: 0.14–0.64, t(42) = 3.03, P = 0.004), suggesting that increased adherence to transparency practices was associated with enhanced clinical utility.

## Discussion

After evaluating 44 CHF RCTs using the van ’t Hooft criteria, several key limitations emerged that collectively undermine the usefulness of these studies for clinicians. Most notably, fewer than 5% of the trials were deemed pragmatic. This low rate reflects trial design features that prioritize explanatory rigor over real-world applicability, such as reliance on placebo controls, narrow eligibility criteria, and highly controlled research settings that may not fully mirror routine CHF care [23, 24].

It is important to emphasize that placebo-controlled randomized trials remain essential for establishing causal treatment effects and represent a cornerstone of evidence-based medicine. Such explanatory trials are designed to maximize internal validity by isolating the effects of an intervention under controlled conditions. However, explanatory trials and pragmatic trials serve complementary purposes. While explanatory trials determine whether an intervention can work under ideal conditions, pragmatic trials help determine whether it works in routine clinical practice. The usefulness framework applied in this study emphasizes pragmatic considerations not to diminish the value of randomized trials, but to evaluate the degree to which published evidence may translate into real-world clinical decision-making.

This is particularly problematic in CHF, a condition marked by complexity and variability [25]. CHF patients often have comorbidities–renal dysfunction, diabetes, or pulmonary hypertension–and their treatment must balance symptom control, polypharmacy, and patient preferences [11, 26, 27]. Yet, some CHF RCTs excluded these subgroups or ignored real-world challenges. Trials rarely assessed outcomes like hospitalization avoidance, functional status, or quality of life—key goals in CHF management [28]. A more useful evidence base would broaden eligibility, consider treatment burdens, and reflect the dynamic decision-making clinicians face.

In addition to limited pragmatism, our review uncovered significant deficits in transparency across CHF trials. Only 13.6% of studies made raw data publicly available, and more than 70% lacked a publicly accessible protocol. These shortcomings impair reproducibility, limit the potential for independent verification, and reduce confidence in the trial’s findings [29]. Perhaps more critically, they constrain the long-term value of these trials by preventing secondary analyses that can inform evolving clinical practice [29]. For clinicians, data sharing is central to building trustworthy, adaptable, and evidence-based guidelines in a rapidly changing field like CHF. A compelling example of the power of data sharing is the 1997 Digitalis Investigation Group (DIG) trial. Initially, the trial demonstrated that digoxin reduced hospitalizations among patients with CHF [30]. However, because the individual patient-level data were made publicly available, at least 41 secondary analyses have since been published [31]. These analyses yielded important sex-specific and dose-related insights, including a 2002 study that revealed an increased mortality risk in women, and a 2003 study showing that elevated serum digoxin levels were associated with harm [32, 33]. These *post hoc* findings played a pivotal role in refining treatment guidelines and informing safer clinical use of the drug. Such real-world impact would not have been possible without open data.

Despite its clear value, data sharing in CHF research remains rare. A recent international survey of cardiology researchers found that 75% cited lack of funding for open access publication as a key barrier, while 20% reported that their institutions placed little emphasis on data accessibility [34]. These findings suggest that structural and cultural disincentives persist, even as journals and funders increasingly endorse transparency. Addressing these obstacles, through data-sharing mandates, reduced publication fees, and institutional incentives, could improve the long-term utility of CHF trials and accelerate the refinement of care for millions of patients.

Economic evaluation is a critical yet underused aspect of CHF trial design. CHF is among the most resource-intensive chronic conditions, with hospitalizations, medications, devices, and long-term care driving major costs [35]. Yet, nearly all trials in our review lacked cost analysis. Tools like value of information (VOI) analysis can help reduce uncertainty about if a new trial is worthwhile [36]. In CHF, where incremental benefits must be balanced with existing standard care, VOI can guide whether to pursue more data or shift to implementation. Budget impact analysis (BIA) estimates the economic effects of adopting new interventions [37]. This is particularly important for CHF treatments, which often include expensive interventions such as SGLT2 inhibitors, implantable cardioverter defibrillators, or advanced CHF therapies [38, 39]. Without a BIA, new interventions may be approved based on clinical outcomes alone, only to face limited uptake due to cost constraints or misaligned resource planning. Despite their utility, VOI and BIA are rare in CHF trials, a missed opportunity to align innovation with sustainability [40, 41]. Wider adoption could promote care that is effective, equitable, and cost-conscious.

### Strengths and limitations

This study systematically reviewed the external validity of RCTs in CHF using the 13-item criteria developed by van ’t Hooft et al, originally designed to assess trials in the context of preterm birth [13]. A major strength of this review was its rigorous methodological approach. The study protocol was preregistered, the search strategy and eligibility criteria were clearly defined in advance, and screening and data extraction were conducted independently and in duplicate to reduce bias. All reviewers underwent structured training and calibration exercises to ensure consistency.

Several limitations should be acknowledged. First, our findings are specific to CHF and may not generalize to other clinical areas. Second, while we assessed trials using predefined pragmatism criteria, we did not account for each trial’s specific inclusion or exclusion criteria, potentially under- or over-representing issues like comorbidity exclusion. Third, although the van ’t Hooft criteria provide structured guidance, some elements (e.g., feasibility or patient-centeredness) involve interpretive judgment. We attempted to minimize subjectivity through duplicate independent assessment, reviewer training, and consensus resolution, but some degree of interpretation is inherent in applying such evaluation frameworks. We mitigated this through consensus among trained reviewers, but interpretation variability remains. Finally, the criteria do not capture all aspects of usefulness, such as implementation feasibility or stakeholder input, which also affect real-world applicability.

### Conclusion

This systematic appraisal highlights substantial gaps in the external validity of RCTs in CHF. Despite their status as the gold standard for evaluating interventions, most CHF trials in our sample lacked pragmatic design features, access to raw data or protocols, and omitted any form of cost-effectiveness analysis. These shortcomings limit the generalizability, transparency, and real-world impact of trial findings. By adopting more inclusive trial designs, prioritizing data sharing, and incorporating economic evaluations such as VOI and BIA, future CHF trials can better serve the needs of clinicians, policymakers, and, most importantly, patients.

## Supplementary Material

Suppl 1 General characteristics of studies included.

## Data Availability

The data supporting the findings of this study have been deposited in Open Science Framework and can be accessed at https://osf.io/ertc9/. No individual participant data were collected, and therefore no additional patient-level data are available.
