# Supplementary material for: Integrating Findings Into Practice: Assessing External Validity of Congestive Heart Failure Trials
Source: Cardiol Res. 2026 Apr 15;17(2):72–81. doi: 10.14740/cr2191 (PMC13094183; doi:10.14740/cr2191)
Supplement: Suppl 1 — General characteristics of studies included. [file cr-17-02-072-s001.docx]

**Supp 1.** General Characteristics of Studies Included

| **Characteristic** | **N = 44** |
| --- | --- |
| Impact Factor of the Journal, Median (IQR) | 3 (2 – 6) |
| Journal Type, n (%) |  |
| Cardiology Journal | 22 (50.0) |
| General Medicine Journal | 17 (38.6) |
| Pharmaceutical / Pharmacology Journal | 3 (6.8) |
| Nephrology Journal | 2 (4.6) |
| Continent of Trial Conduct, n (%) |  |
| Asia | 21 (47.7) |
| Europe | 8 (18.2) |
| North America | 7 (15.9) |
| Multiple | 6 (13.6) |
| Africa | 1 (2.3) |
| Australia | 1 (2.3) |
| Sample, Median (IQR) | 82 (52.0 – 191) |
| Funding Source, n (%) |  |
| Private/Industry | 17 (38.6) |
| Funding Not Reported | 12 (27.3) |
| Government | 5 (11.4) |
| Multiple Funding Types | 5 (11.4) |
| University | 3 (6.8) |
| Self-funded/No Outside Funding | 2 (4.5) |
| Year, n (%) |  |
| 2023 | 3 (6.8) |
| 2022 | 2 (4.5) |
| 2021 | 4 (9.1) |
| 2020 | 3 (6.8) |
| 2019 | 1 (2.3) |
| 2018 | 5 (11.4) |
| 2017 | 4 (9.1) |
| 2016 | 7 (15.9) |
| 2015 | 6 (13.6) |
| 2014 | 8 (18.2) |
| 2013*** | 1 (2.3) |

***ePublication date from 2013, with in-print date in 2014.
